# CircMED13L_012 promotes lung adenocarcinoma progression by upregulation of MAPK8 mediated by miR-433-3p

**DOI:** 10.1186/s12935-021-01811-4

**Published:** 2021-02-16

**Authors:** Wenshu Chen, Guanying Zheng, Jianyuan Huang, Lihuan Zhu, Wujin Li, Tianxing Guo, Yangyun Huang, Xiaojie Pan

**Affiliations:** 1 Department of Thoracic Surgery, Shengli Clinical Medical College of Fujian Medical University, Fujian Provincial Hospital, No. 134 East Street, 350001 Fuzhou, China; 2 Department of Pulmonary and Critical Care Medicine, Shengli Clinical Medical College of Fujian Medical University, Fujian Provincial Hospital, 350001 Fuzhou, China

**Keywords:** NSCLC, circMED13L_012, MAPK8

## Abstract

**Background:**

Metastasis and disease refractoriness remain as major challenges for non-small cell lung cancer (NSCLC) treatment and understanding the underlying molecular mechanisms is of scientific and clinical value. Therefore, in this study, we aimed to explore the effects of circMED13L_012 on the proliferation, migration, invasion and drug-resistance of NSCLC tumor cells.

**Methods:**

In this study, we utilized clinical samples and NSCLC cell lines to explore the association between circMED13L_012 expressions and tumor cell metastasis and chemo resistance. CCK8 and transwell assay were conducted to explore the impact of circMED13_012 on NSCLC tumor proliferation and migrative capabilities. Dual-luciferase reporter gene assay was conducted to validate the circMED13L_012 interaction network.

**Results:**

Our results demonstrated that circMED13L_012 exhibited significantly elevated average level in our clinical samples of NSCLC, compared with normal tissues. circMED13L_012 level was positively correlated with disease stage and metastatic status. Increased circMED13L_012 expression was associated with the enhanced migration, proliferation and chemo resistance of NSCLC cell lines. Further experiments indicated that circMED13L_012 promoted malignant behavior of NSCLC tumor cells by targeting MAPK8 through modulation miR-433-3p expression.

**Conclusions:**

Our study for the first time demonstrated that circMED13L_012–miR-433-3p–MAPK8 axis played important role for NSCLC pathogenesis, which could be potential therapeutic target for the development of future NSCLC treatment.

## Background

Lung cancer is one of the most dangerous diseases worldwide with a high mortality and morbidity [1]. Up to date, lung cancer refractoriness to standardized immunochemotherapeutic strategies and disease metastasis have become serious challenges for the treatment of non-small cell lung cancer (NSCLC) patients [2]. It has been reported that for advanced stage NSCLC patients, more than 1/5 of the patients suffered from disease refractoriness [3]. Therefore, it is of scientific and clinical significance to fully understand the underlying molecular mechanism of NSCLC refractoriness and tumor metastasis.

Circular RNAs (circRNAs) are members from small RNA family which function as molecular sponges and by direct binding they were able to modulate the expression of a range of microRNAs [4, 5]. Current researches demonstrated that circRNAs were involved in the pathogenesis of multiple malignant diseases including colorectal cancer [6], hepatocellular carcinoma [7], breast cancer [8] and gastric cancer [9]. And another research demonstrated that tumor epithelial to mesenchymal transition (EMT) process was also closely associated with circRNAs biogenesis [10], which indicating potential roles of circRNAs in tumor metastasis.

However, up till now, the exact role of circMED13_012 in lung cancer pathogenesis and disease progression remains undiscovered. Therefore, in this study, we aimed to evaluate the molecular impact of circMED13_012 on lung cancer cells and further explore the gene regulatory network of circMED13_012 for NSCLC.

## Materials and methods

### Patient recruitment and sample collection

A total of 180 patients diagnosed with NSCLC in our cancer center receiving surgical treatment during Jan 2019 to Dec 2019 were included in our study. 120 patients’ tumor biopsy samples and adjacent normal tissues were acquired. Tissues were stored immediately after surgical resection using liquid nitrogen. Informed consent was obtained for all patients enrolled in this study. And our study was approved by Ethical Committee of Fujian Provincial Hospital.

## Cell line culturing

NSCLC cell line NCI-H1299, H1975, SPC-A-1, A549 and normal human brochial epithelial cell line HNBE were purchased from American Type Culture Collection (ATCC; Manassas, VA, USA). Cells were cultured using RPMI-1640 medium with 10% fetal bovine serum (FBS; Hyclone, South Logan, UT, USA). And 150 IU/mL penicillin combined with 150 µg/mL streptomycin (Invitrogen, Carlsbad, CA, USA) were added during cell culture, with the settings of environmental parameters as 37°C, 5% CO2.

## Cell transfection

NCI-H1299 and A549 cells were placed into 8-well plates with density of 1 × 10^6^ cells per well. Lipofectamine 2000 (Invitrogen, Carlsbad, CA, USA) was used for transfection of vectors including circMED13L_012 overexpression plasmid, miR-433-3p mimics and inhibitor, sh- circMED13L_012, MAPK8 siRNAs according to the instructions of manufacturer.

## Real time qRT-PCR quantification

TRIzol agent (Invitrogen, Thermo Fisher Scientific, Inc) was used for RNA extraction according to the standardized protocol. cDNA was collected through reverse transcription for subsequent qRT-PCR experiments. qRT-PCR reaction condition was set as 94°C for 30s, 55°C for 30s, 72°C for 90s, with total cycles of 45. Primers used in this study include: MED13L primers forward 5′-TGCTGTCAGAGCAAGCGAG − 3; reverse 5′-GATCGCTTTGAAGAGCAGCG-3′; U6 forward, 5′-CTCGCTTCGGCAGCACAT-3′; reverse, 5′-AACGCTTCACGAATTTGCGT-3; miR-433-3p forward, 5′- GGAGAAGTACGGTGAGCCTG − 3′; reverse, 5′- CAGTGCGTGTCGTGGAGT − 3′; Actin forward, 5′-GTCCACCGCAAATGCTTCTA-3′; reverse, 5′-TGCTGTCACCTTCACCGTTC-3′.

## Western blot

RIPA agent (Beyotime, Shanghai, China) was used to extract protein from the clinical samples and proteins were quantified by BCA assay. Sodium dodecy lsulfate–polyacrylamide gel electrophoresis was used to separate proteins and then proteins were transferred to polyvinylidene difluoride (PVDF) membranes (Millipore, Billerica, MA, USA). The membranes were incubated at 4 °C overnight with primary antibody (Cell Signaling Technology, Danvers, MA, USA), after subsequently rinsing with the tris-buffered salin and Tween buffer solution (TBST; Sigma-Aldrich, St. Louis, MO, USA), membranes were then incubated with the secondary antibody at room temperature for 1 h. Chemiluminescence was used to expose the protein bands on the membrane.

## Cell proliferation CCK8 assay

Prior to experiments, cells from each treatment/control group were inoculated into 96-well plates with a density of 1 × 10^3^ cells/well. Cell Counting Kit-8 (CCK-8; Dojindo Molecular Technologies, Kumamoto, Japan) solution (10 µL) was added to each well after 1, 2, 3, 4 and 5 days, respectively. Afterwards cells were incubated at 37 °C for 1 h. The absorbance was recorded of each well at 450 nm by a microplate reader.

## Cell survivability and migration assay

2 × 10^6^ Cells for each group were treated for 4 h with a series of dosage of chemoagents including docetaxel, doxorubicin, gefitinib. Then cells were washed with cold PBS before incubation with 75% ethanol at −20 °C overnight. Cells were stained with 10 µL of propidium iodide (PI) and Annexin V-FITC (Thermo Fisher Scientific) for 20 min at room temperature, and flow cytometry (FACScan™, BD Biosciences, Franklin Lakes, NJ, USA) analysis was performed for evaluation of cellular apoptosis. 6 × 10^4^ transfected cells were put into the upper chamber (8-µm) (Corning, Lowell, MA, USA). As for the bottom chamber, the medium with 10% fetal bovine serum (FBS; Gibco, Grand Island, NY, USA) was added. Cells for each experimental group were respectively incubated under 37 °C, 5% CO2 condition for 48 h. Penetrating cells were fixed in 70% ethanol for 40 min and stained with 0.1 % crystal violet for 15 min. Five randomly selected fields for each sample was selected for the number calculation of penetrating cells.

## RNA-immunoprecipitation

Nuclear proteins of cells were extracted according to the manufacturers’instructions. 10 % of the total nuclear protein was used as input control. Anti-IgG and anti-Ago2 antibodies were then used to incubate with the remaining protein at 4 °C for 2 h, and protein was subsequently incubated with protein A/G plus-agarose at 4°C overnight. The proteins were then centrifuged at 4°C, 2000 r/min for 1 min. The precipitate was re-suspended in NETN100. 10 % of precipitate, and input control and IgG sample were used for protein level quantification. The remaining samples were used for RNA isolation, purification and identification.

## Dual‐luciferase reporter gene assay

Prior to experimental procedures, cells were digested using trypsin. Then cells were inoculated into 24-well plates before transfection. Transfection reagents were prepared as described below: Tube A: circMED13L_012-WT plasmid and circMED13L_012-MUT plasmid or MAPK8-WT/MUT plasmid were mixed with culture medium; Tube B: miR-433-3p-WT plasmid and miR-NC plasmid were mixed with culture medium; Tube C: transfection reagent was mixed with culture medium. Tube C mixture was separately added into Tube A and B. Mixtures in Tube A and B were added in each well and incubated for 48 h. Transfection efficiency was observed by a fluorescence microscope (Leica, Wetzlar, Germany).

### Statistical analysis

Statistical Product and Service Solutions (SPSS) 22.0 (Chicago, IL, USA) was applied for all statistical analysis. GraphPad Prism software (Version X; La Jolla, CA, USA) was introduced for figure processing. Experimental data were expressed as mean ± standard deviation (SD). Results among more than 2 groups was analyzed using one-way analysis of variance (ANOVA). Student’s t test was applied to analyze data between 2 groups. p < 0.05 was considered statistically significant.

## Results

### Characterization of circMED13L_012 and its expression pattern in NSCLC samples

**Fig. 1:**
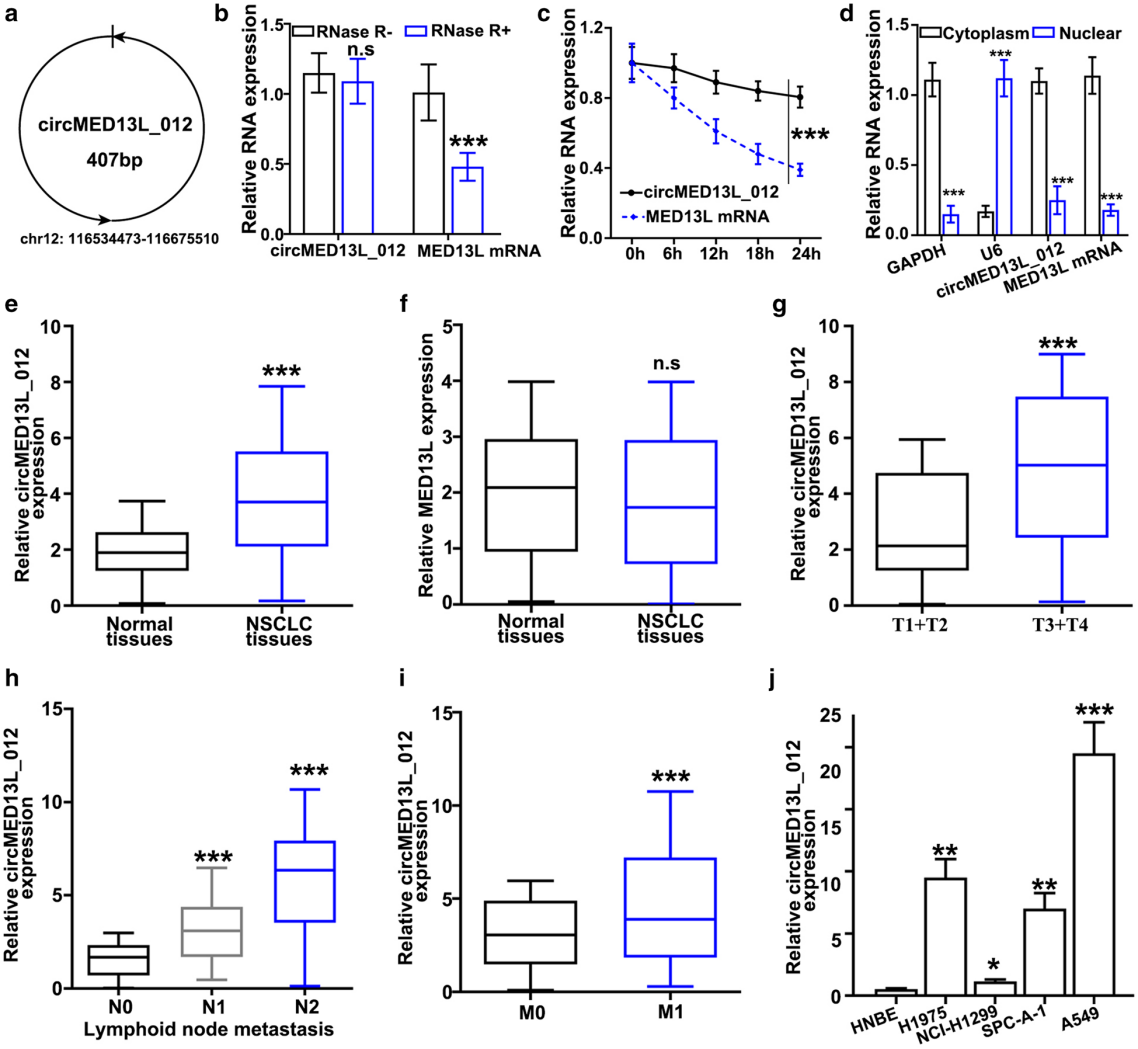
Characterization of circMED13L_012 and its expression pattern in NSCLC samples. **a** Schematic illustration of chromosomal location of circMED13L_012. **b** qRT-PCR analysis of circMED13L_012 and MED13L mRNA level in A549 cells which were treated by RNase or negative control respectively. **c** qRT-PCR analysis of circMED13L_012 and MED13L mRNA level in A549 cells which were treated by actinomycin-D or negative control respectively for different period of time (0 h, 6 h, 12 h, 18 h, 24 h). **d** relative expression level detection of circMED13L_012, MED13L, GAPDH and U6 in cytoplasmic or nuclear region of A549 cells. **e, f** Comparison of circMED13L_012 and MED13L expression level between NSCLC and matched normal lung tissues. **g** Comparison of circMED13L_012 expression level between early stage (T1, T2) and advanced stage (T3, T4) NSCLC tumors samples. **h** Comparison of circMED13L_012 expression level in NSCLC tumor samples with or without lymph node metastasis. **i** Comparison of circMED13L_012 expression level in NSCLC tumor samples with or without distal organ metastatic disease. **j** Comparison of circMED13L_012 expression level among different NSCLC cell lines (NCI-H1975, NCI-H1299, A549, SPC-A-1) and human normal bronchial epithelial cell line HNBE

As shown in Fig. 1a, CircMED13L_012 was located at Chromosome 12: 116,534,473–116,675,510, with a length of 407 bp. RNase treatment experiment indicated the relatively high structural stability of circMED13L_012, compared with MED13L mRNA (Fig. 1b). Further experiments using actinomycin D, which is a transcription inhibitor, indicated that circMED13L_012 circular transcripts were more stable compared with linear transcripts of MED13L mRNA (Fig. 1c). In addition, by qRT-PCR method, we further compared the expression level of MED13L mRNA and circMED13L_012 in cellular cytoplasmic and nuclear region. As a result, MED13L mRNA and circMED13L_012 were shown to be significantly enriched in cytoplasm (Fig. 1d).

Then, in order to understand the association between circMED13L_012 and disease status in NSCLC patients, we performed qRT-PCR study to detect circMED13L_012 expression value in NSCLC and matched normal tissue samples. Our study demonstrated that circMED13L_012 expression levels were significantly higher, in comparison with normal tissues (Fig. 1e). While there is no significantly different level of MED13L mRNA expression level between NSCLC tumor samples and matched normal tissues (Fig. 1f). Furthermore, circMED13L_012 levels among NSCLC tumor samples of different stages were compared and results indicated that advanced-stage NSCLC patients (stage III-IV) showed significantly higher level of circMED13L_012 expression level in comparison with early stage tumor samples (stage I/II) (Fig. 1g). As for samples of patients with advanced stage of lymphoid node metastatic diseases (N2), they also exhibited significantly higher level of circMED13L_012 comparing with patients in N1 or N0 status. Meanwhile, N1 patients also showed significantly higher that no status patients. (Fig. 1h). Consistently, patients with distal organ metastatic disease (M1) also demonstrated increased circMED13L_012 level in their tumor cells, comparing with patients without distal organ metastasis (M0) (Fig. 1i). Next, qRT-PCR detection of circMED13L_012 expression levels also indicated that circMED13L_012 levels in several NSCLC cell lines (NCI-H1299,H1975, SPC-A-1, A549) were significantly higher compared to normal human bronchial epithelial cell line HNBE (Fig. 1j).

### Effects of circMED13L_012 on the malignant behavior of NSCLC cell lines

**Fig. 2:**
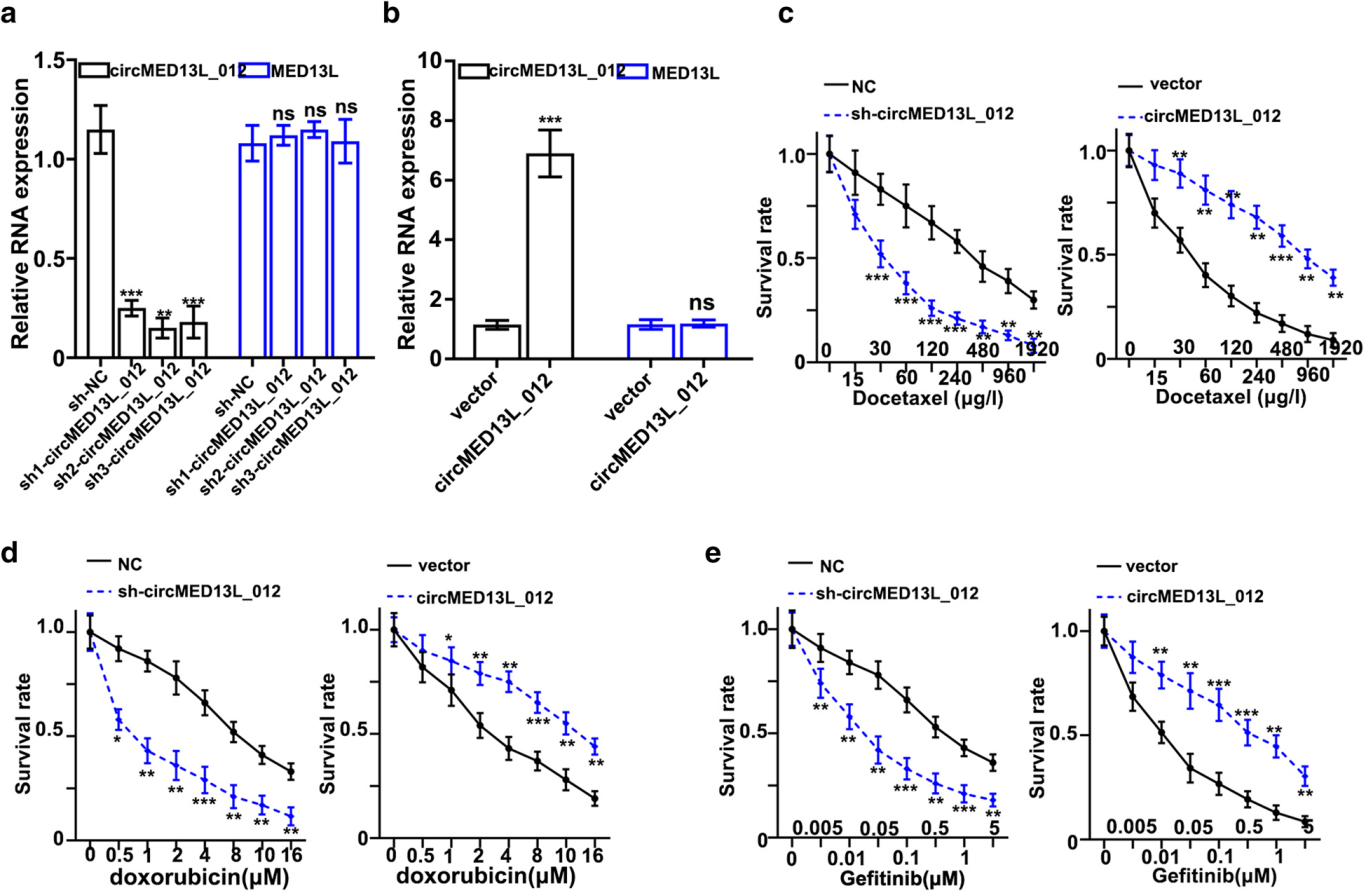
Influence of circMED13L_012 on the malignant behavior of NSCLC cell lines. **a **Impact of circMED13L_012 specific shRNAs on expression level of circMED13L_012 and MED13L_012 in A549 tumor cells.** b **qRT-PCR study of circMED13L_012 and MED13L expression level in A549 tumor cell groups treated by circMED13L_012 overexpression vector and control vector respectively. **c–e **Tumor survivability test of A549 cell groups transfected by sh-circMED13L_012, circMED13L_012 overexpression vector and control vector respectively. Tumor cells were challenged by docetaxel (**c**), doxorubicin (**d**) and gefitinib (**e**), with different range of concentrations for each chemo-agent

To further investigate the role of circMED13L_012 played in NSCLC pathogenesis, in this study we utilized three different circMED13L_012-specific shRNAs and circMED13L_012 over-expression vector to modulate circMED13L_012 expression. As shown in Fig. 2a and b, all three shRNAs and circMED13L_012 over-expression vector exhibited significant suppressive and promotive effects on circMED13L_012 level, while they had no influences on the mRNA expression level of MED13L. Next, we evaluated the effects of circMED13L_012 expression modulation on tumor cell survivability under treatment of several chemotherapeutic agent including docetaxel, doxorubicin and gefitinib. By transfection of sh- circMED13L_012 and circMED13L_012 overexpression vector into A549 cells, our study results indicated that cells transfected with sh-circMED13L_012 exhibited significantly suppressed chemo-resistance. In the meantime, cells transfected with circMED13L_012 over expression vector demonstrated significantly promoted survivability compared with normal control (Fig. 2c–e).

**Fig. 3:**
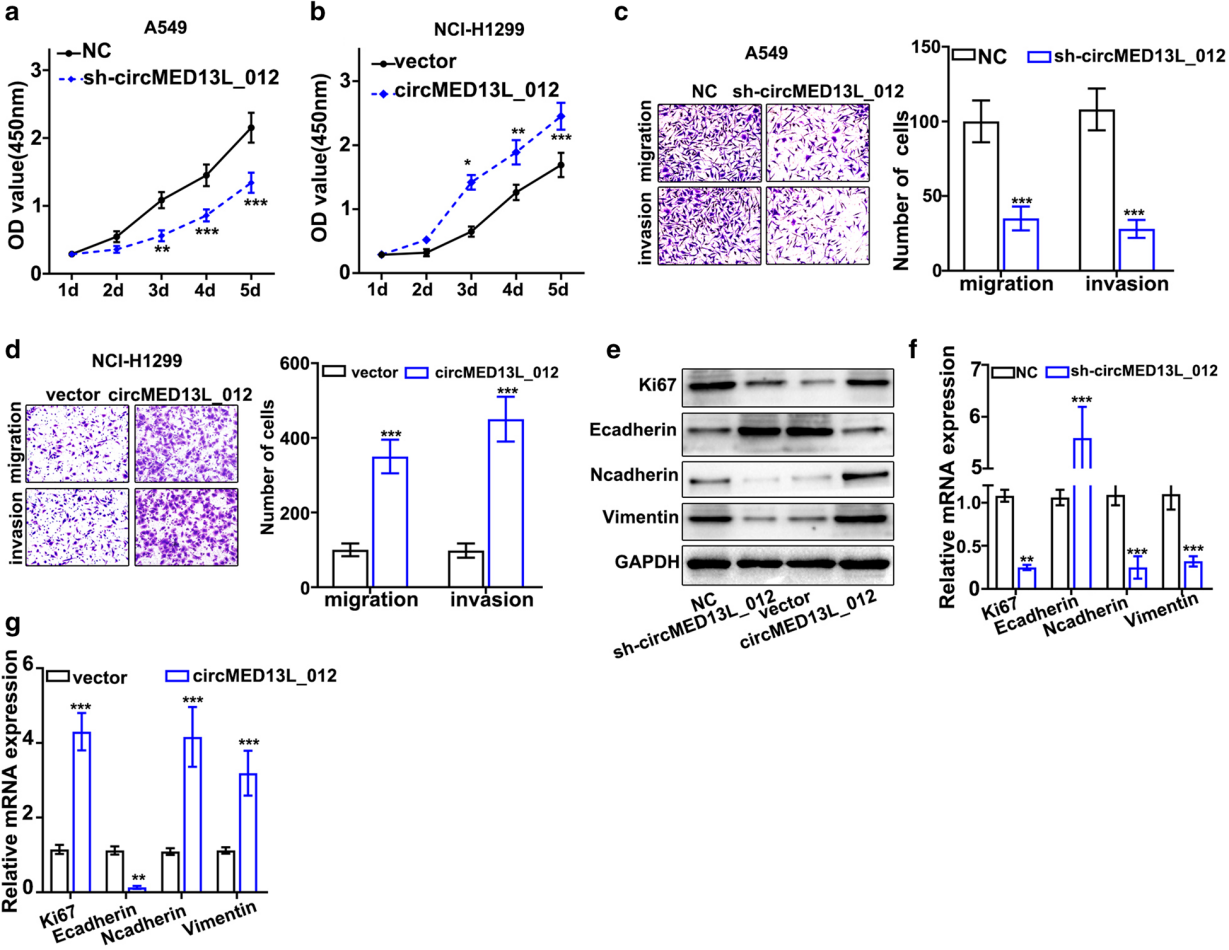
Influence of circMED13L_012 modulation on the proliferative abilities of NSCLC cell lines. **a**, **b** Influences of sh-circMED13L_012 or circMED13L_012 overexpression vector transfection on tumor cell proliferation. A549 and NCI-H1299 cells were respectively transfected with sh- circMED13L_012, circMED13L_012 overexpression and control vectors for 1d-5d and CCK8 analysis was performed on each cell group, **c**, **d** A549 and NCI-H1299 cells were respectively transfected with sh-circMED13L_012, circMED13L_012 overexpression vector or control vectors, Transwell analysis was performed on each cell group to evaluate the influences of circMED13L_012 overexpression and silencing on the migration and invasion of the tumor cells. **e **Western Blot analysis on Ki67, E-cadherin, N-Cadherin and vimentin protein expression values in A549 cells transfected by sh-circMED13L_012, circMED13L_012 overexpression vector and control vector respectively, **f**, **g** qRT-PCR study on Ki-67, E-Cadherin, N-Cadherin and vimentin mRNA expression values in A549 cells transfected by sh-circMED13L_012, circMED13L_012 overexpression vector and control vector respectively

Then, we evaluated the influences of circMED13L_012 modulation on the proliferative abilities of NSCLC cell lines. As shown in Fig. 3a and b, sh-circMED13L_012 treated A549 cells exhibited significantly decreased cell proliferation compared with control group, while circMED13L_012 overexpression in NCI-H1299 significantly promoted cellular proliferation. In addition, after treatment with sh-circMED13L_012, the migration and invasion ability of A549 cells were remarkably suppressed (Fig. 3c). However, circMED13L_012 overexpression significantly promoted the cell migration and invasion of NCI-H1299 cells (Fig. 3d). The protein levels of Ki67, N-Cadherin, and vimentin were remarkably suppressed by sh-circMED13L_012, but the expression of E-Cadherin was significantly increased (Fig. 3e, f). However, circMED13L_012 overexpression exerted opposite influence on the levels of Ki67, E-Cadherin, N-Cadherin, and vimentin (Fig. 3g). circMED13L_012 overexpression significantly increased the levels of Ki67, E-Cadherin, and vimentin, but inhibited N-Cadherin. The results demonstrated that circMED13L_012 over expression was significantly related with enhanced cellular expression of Ki67, vimentin and N-cadherin and suppressed expression of E-Cadherin.

## circMED13L_012 modulates miR-433-3p expression

**Fig. 4:**
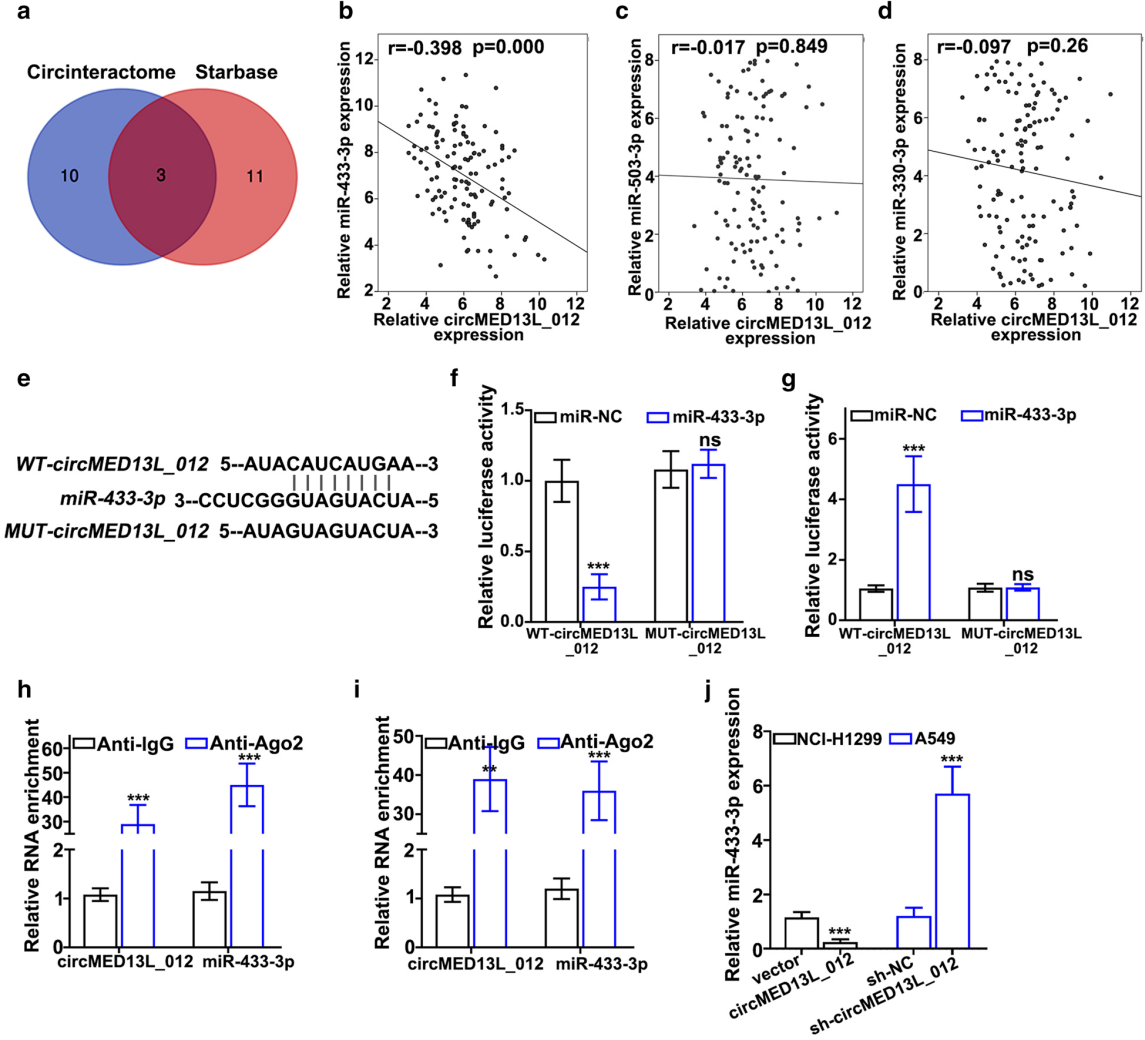
circMED13L_012 modulates miR-433-3p expression. **a** Bioinformatic prediction of circMED13L_012 interacting miRNAs using Circinteractome and Starbase online database platform. **b**–**d** Association of circMED13L_012 expression level and miR-433-3p, miR-503-3p, miR-330-3p expression level in NSCLC tumor samples. **e** Design of wildtype and mutated circMED13L_012 vector and predicted binding location with miR-433-3p. **f**,** g** Dual luciferase reporter gene assay via wildtype or mutated circMED13L_012 vector transfection with or without treatment of sh-circMED13L_012 to validate the binding of miR-433-3p with wildtype circMED13L_012. **h**,** i.** RNA immunoprecipitation assay to detect the binding of circMED13L_012 and miR-433-3p with anti-Ago2 antibody, in comparison with control IgG antibody in A549 and NCI-H1299 tumor cells. **j.** qRT-PCR evaluation of the impact of circMED13L_012 overexpression and silencing on expression level of miR-433-3p in NCI-H1299 and A549 cells.

As it is well known that circRNAs regulate miRNAs expression by acting as molecular sponges through interaction with RNA-induced silencing complex (RISC). In order to further explore the detailed molecular mechanism of circMED13L_012, we performed bioinformatic prediction of circMED13L_012 interacting miRNAs. As shown in Fig. 4a, by cross-checking prediction results of two circRNA-miRNA interaction online platforms (Circinteractome, https://circinteractome.irp.nia.nih.gov and Starbase, http://starbase.sysu.edu.cn), we identified a total of 3 miRNAs (miR-433-3p, miR-503-3p and miR-330-3p) that potentially interacted with circMED13L_012. Further validation analysis on NSCLC clinical samples demonstrated that only miR-433-3p expression level was negatively correlated with circMED13L_012 expression which reached statistical significance (r=-0.398, *P* = 0.000) (Fig. 4b and d). By prediction of binding site of circMED13L_012 with miR-433-3p (Fig. 4e), we performed dual-luciferase reporter gene assay *in vitro* using vectors carrying wildtype and mutated circMED13L_012 and miR-433-3p or control. Our results indicated that luciferase activity of miR-433-3p was significantly suppressed for A549 and NCI-H1299 tumor cells transfected by wildtype circMED13L_012 vector, in comparison with cell group transfected with mutated circMED13L_012 vector (Fig. 4f). While cell group with wild type-circMED13L_012 vector treated with sh-circMED13L_012 exhibited significantly increased miR-433-3p level, in comparison with mutated circMED13L_012 vector group (Fig. 4g). RNA immunoprecipitation (RIP) experiments using Ago2 antibody demonstrated that both circMED13L_012 and miR-433-3p were significantly enriched with Ago2 (Argonaute) antibodies compared with control IgG (*P* < 0.001) (Fig. 4h and i), indicating their functions were closely associated with RISC. Moreover, circMED13L_012 expression modulation via sh-circMED13L_012 and circMED13L_012 overexpression vector presented significant influences on miR-433-3p treatment (*P* < 0.001) (Fig. 4j).

### circMED13L_012 modulates miR-433-3p and exerts its biological effects by interacting with MAPK8 gene

**Fig. 5:**
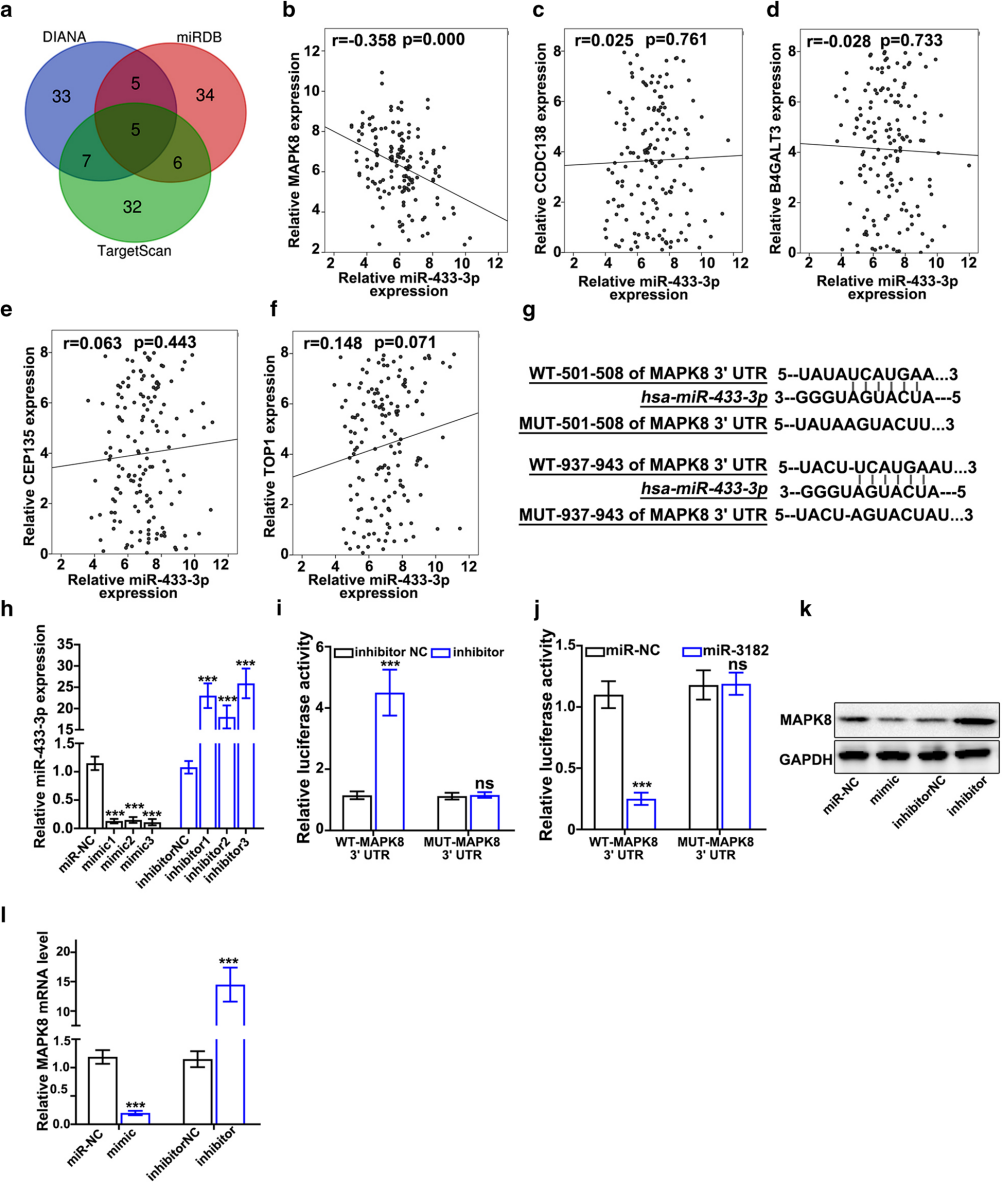
circMED13L_012 modulates miR-433-3p through affecting MAPK8.** a** miR-433-3p target gene prediction based on three microRNA-gene interaction databases including DIANA, miRDB and TargetScan.** b**–**f** qRT-PCR study exploring the association of miR-433-3p expression level and MAPK8 (B), CCDC138 (C), B4GALT3 (D), CEP135 (E), TOP1 (F) mRNA expression level in NSCLC clinical samples.** g** Prediction of miR-433-3p binding site with wildtype MAPK8 3’UTR region and design of vector including mutated MAPK8 3’UTR region.** h** qRT-PCT validation of the impact of miR-433-3p specific mimics and inhibitor on miR-433-3p expression.** i**,** j** Dual luciferase reporter gene assay to evaluate modulative effects of miR-433-3p specific inhibitor (I) and mimics (J) on MAPK8 expression.** k.** Western blot analysis on the modulative effects of miR-433-3p specific inhibitor and mimics on the MAPK8 protein expression level

In order to explore the detailed mechanism of circMED13L_012 - miR-433-3p regulatory axis, we performed targets gene prediction of miR-433-3p via online miRNA-gene interacting platforms including DIANA, TargetScan and miRDB, and a total of five genes (MAPK8, CCDC138, B4GALT3, CEP135 and TOP1) as potential target of miR-433-3p were identified for all three databases (Fig. 5a). qRT-PCR validation on NSCLC clinical samples indicated that only MAPK8 gene was shown to be negatively correlated with miR-433-3p expression level, which was statistically significant (Fig. 5b and f) (r=-0.358, *P* = 0.000). To confirm the interaction of miR-433-3p and MAPK8 gene, two different miR-433-3p binding sites of MAPK8 3’UTR region were identified and vectors carrying WT-MAPK8-3’UTR and MUT-MAPK8-3’UTR regions were generated (Fig. 5g). miR-433-3p mimics / specific inhibitors were also constructed and their modulation effects on miR-433-3p expression were also evaluated (Fig. 5h). Dual-luciferase reporter gene assay was also performed on A549 and NCI-H1299 cell lines transfected with miR-433-3 mimics / specific inhibitors, in combination of vectors carrying WT-MAPK8-3’UTR and MUT-MAPK8-3’UTR region, respectively. Experiments results demonstrated that miR-433-3 specific inhibitors significantly increased luciferase activity in cells transfected with WT-MAPK8-3’UTR vector compared with cells transfected with MUT-MAPK8-3’UTR (Fig. 5i). Additionally, treatment with miR-433-3 mimics significantly suppressed luciferase activity in cells with WT-MAPK8-3’UTR vectors (Fig. 5j). While no significant differences in luciferase activities were shown in cell group with MUT-MAPK8-3’UTR vector. Consistently, western-blot and qRT-PCR study results also confirmed the modulative activities of miR-433-3 mimics / specific inhibitors on MAPK8 protein/mRNA expression (Fig. 5k and l).

**Fig. 6:**
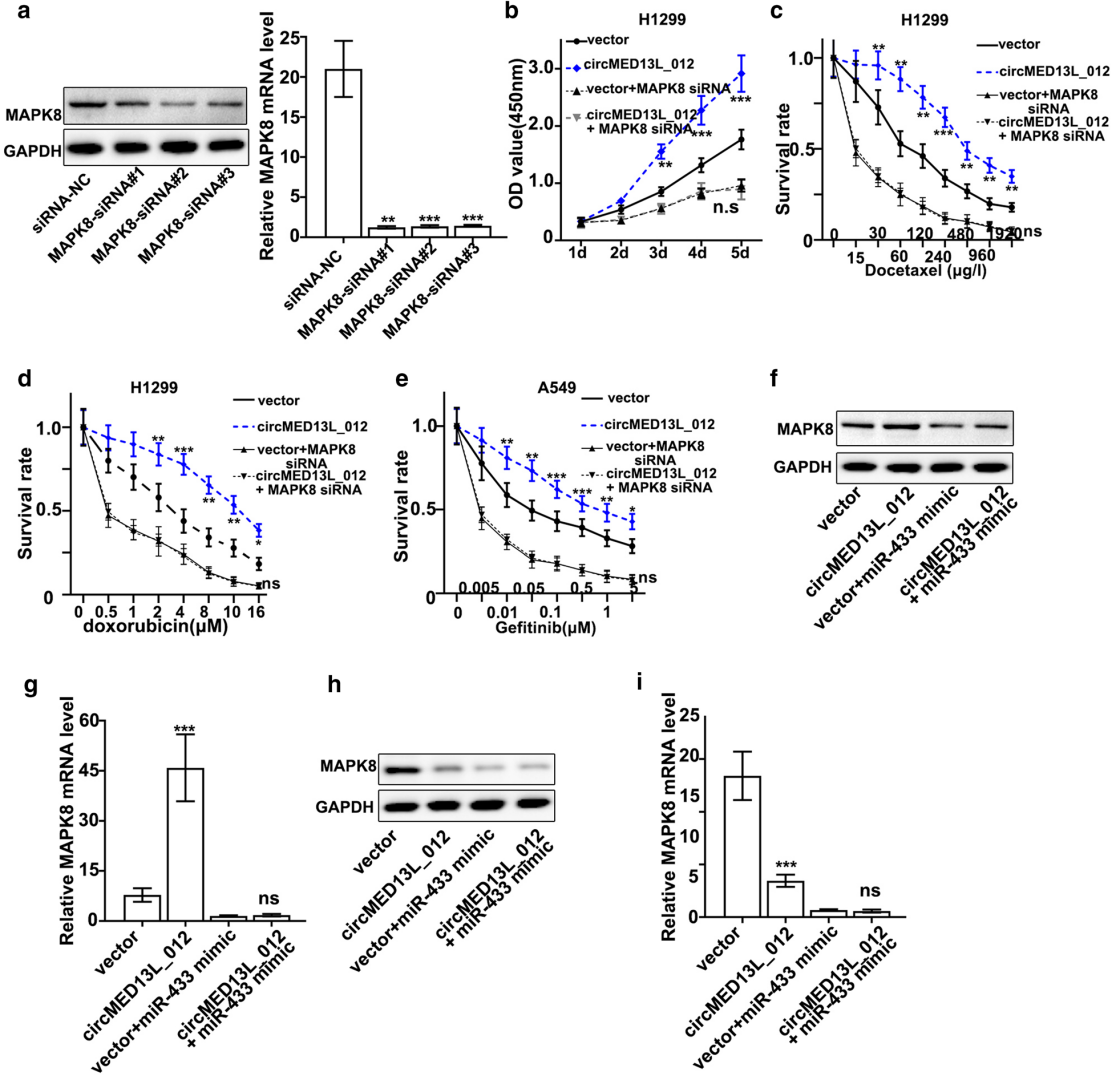
Function identification of circMED13L_012-miR-433-3p–MAPK8 axis in NSCLC cells.** a** Western Blot and qRT-PCR validation of the effects of MAPK8 specific siRNAs treatment on the protein and mRNA level of MAPK8 in A549 cells.** b** Cell proliferation analysis on NCI-H1299 cells transfected by circMED13L_12 overexpression vector, with or without MAPK8 siRNA.** c**–**e** Cell survivability test of NCI-H1299 and A549 cells which were transfected by circMED13L_12 overexpression vector, with or without MAPK8 siRNA. Cell groups were challenged by three different chemo-agents with a range of dosages respectively, i.e. docetaxel, doxorubicin and gefitinib.** f** Western blot analysis on A549 cells transfected by circMED13L_12 overexpression vector, with or without miR-433-3p mimics.** g** qRT-PCR study on A549 cells transfected by circMED13L_12 overexpression vector, with or without miR-433-3p mimics.** h** Western blot analysis on NCI-H1299 cells transfected by sh-circMED13L_12 vector, with or without miR-433-3p mimics.** i** qRT-PCR study on NCI-H1299 cells transfected by sh-circMED13L_12 vector, with or without miR-433-3p mimics

Finally, to validate the biological effects of circMED13L_012-miR-433-3p–MAPK8 axis in NSCLC cells identified in this study, we designed MAPK8-specific siRNAs and validated their suppressive effects on MAPK8 mRNA/protein expression (Fig. 6a). Cell proliferation assay and performed on NCI-H1299 cells transfected by MAPK8 siRNA with or without circMED13L_012 overexpression vectors indicated that MAPK8 siRNAs abrogated circMED13L_012 promotive effects on cellular proliferation (Fig. 6b). Cellular survival experiments were also performed on A549 and NCI-H1299 cells treated with different concentrations of docetaxel, doxorubicin and gefitinib (Fig. 6c–e). Results indicated that MAPK8 siRNAs also abrogated the increased cellular survivability caused by circMED13L_012 overexpression. Western Blot and qRT-PCR experiments also confirmed that by transfection of circMED13L_012 overexpression vector, MAPK8 protein and mRNA level was significantly elevated, while such effects were abrogated by co-transfection of miR-433-3p mimics (Fig. 6f and g). On the other hand, sh-circMED13L_012 generated suppressive effects on MAPK8 mRNA and protein expression, and its suppressive effects were further elevated by co-transfection of miR-433-3p mimics (Fig. 6h and i).

## Discussion

As it is well acknowledged that NSCLC is a genetically heterogeneous disease, multiple signaling pathways involving cell proliferation and survival play important parts in NSCLC pathogenesis. Among them, MAPK pathway has been demonstrated as important component in NSCLC pathogenesis [11]. Genetic mutations of several key kinases along MAPK pathways including RAS, RAF, MEK and ERK have been indicated to account for the increased tumor proliferative abilities as well as survival and chemo-resistance. For example, KRAS mutation was found in 30 % of lung adenocarcinoma patients [12], while BRAF mutation was less frequent. MEK1 and MEK2 were known as downstream components of KRAS/RAF signaling pathway, and current studies have shown they were potential therapeutic targets for NSCLC and specific inhibitor, Trametinib has been developed and exhibited promising effects in clinical trials [13–15].

On the other hand, MAPK 8/9/10 (also known as JNK 1/2/3) are also components in MAPK pathway [16] but their functions in NSCLC were not fully understand. Recent study indicated that MAPK8/9/10 activation via Rabl3 depletion was associated with enhanced autophagy for lung cancer cells [17], this phenomenon was consistent with previous findings that MAPK signaling pathway activation was associated with autophagy enhancement in ovarian and gastric cancer cells [18, 19]. Therefore, increased tumor chemo-resistance of NSCLC caused by MAPK8 expression elevation could possibly be explained by enhanced tumor cellular autophagy, once more mechanistic studies were performed to further confirm such putative link.

In this study, we demonstrated for the first time that circMED13L_012 regulated MAPK8 expression level by reducing miR-433-3p level in NSCLC patients. Currently, little is known about the molecular function and its pathological significance of circMED13L_012 in NSCLC. However, previous studies did find that MED13L gene played interesting part in the radio-sensitivity of NSCLC tumor cells, as radiation triggers silencing of MED13L and MED13L suppression decreased their physical interactions and reduced recruitment of acetyltransferase P300 to chromatin via Mediator complex, and they in turn suppressed the activities of multiple oncogenes [20]. In this study, although we found no significant association of circMED13L_012 expression with MED13L mRNA level, the potential interactions of circMED13L_012 with Mediator complex in NSCLC pathogenesis required further investigation.

Moreover, our study presented consistent results of the suppressive effects of miR-433-3p on tumor cell proliferation. Previous researches have demonstrated that miR-433-3p attenuated glioma cell growth and invasion/migration by targeting CREB [21]. Besides, another study indicated that for esophageal squamous cell carcinoma, miR-433-3p overexpression also exhibited inhibitory effects on tumor cells proliferation, invasion, and migration. And study also demonstrated that growth factor receptor-bound protein 2 (GRB2) is the target of miR-433-3p that mediated the tumor inhibitory function of miR-433-3p [22]. Additionally, miR-433-3p were also shown to possess tumor inhibitory effects in osteosarcoma [23], breast cancer [24], bladder cancer [25], renal carcinoma [26]. As for NSCLC, previous studies also showed that miR-433-3p targeted WT1 associated protein (WTAP) to reduced tumor cell proliferation and migration. Therefore, based on previous findings and our study results, miR-433-3p could be valuable therapeutic target in future NSCLC treatment, once further detailed studies could be performed to fully unveil its role in NSCLC pathogenesis and disease progression. Therefore, therapeutic strategies inhibiting circMED13L_012 or MAPK8 and promoting miR-433-3p could be used in the future for NSCLC patient treatment.

There are some limitations in this study. Firstly, it is better to perform xenograft tumors experiments in vivo to confirm the role of circMED13L_012/miR-433-3p/MAPK8 in regulating tumors. Secondly, influence of circMED13L_012/miR-433-3p/MAPK8 on the apoptosis and cell cycle of tumor cells was missing in this study.

## Conclusions

Our study demonstrated for the first time that circMED13L_012 played unique part in promotion NSCLC tumor cell proliferation, invasion and chemo resistance. circMED13L_012 could be valuable predictive biomarker for NSCLC prognosis and metastasis evaluation. And circMED13L_012-miR-433-3p-MAPK8 axis presented in our study could be novel therapeutic target for future NSCLC treatment.

## Data Availability

The datasets used and analyzed in the current study are available from the corresponding author in response to reasonable requests.

## Abbreviations

circRNAs: Circular RNAs
EMT: Epithelial to mesenchymal transition
PVDF: Polyvinylidene difluoride
PI: Propidium iodide
RISC: RNA-induced silencing complex
RIP: RNA immunoprecipitation
WTAP: WT1 associated protein

## References

[1] Siegel RL, Miller KD, Jemal A (2020). Cancer statistics, 2020. Cancer J Clin.

[2] Popper HH (2016). Progression and metastasis of lung cancer. Cancer Metastasis Rev.

[3] Liu WJ, Du Y, Wen R, Yang M, Xu J (2020). Drug resistance to targeted therapeutic strategies in non-small cell lung cancer. Pharmacol Ther.

[4] Hansen TB, Jensen TI, Clausen BH, Bramsen JB, Finsen B, Damgaard CK, Kjems J (2013). Natural RNA circles function as efficient microRNA sponges. Nature.

[5] Guo JU, Agarwal V, Guo H, Bartel DP (2014). Expanded identification and characterization of mammalian circular RNAs. Genome biology.

[6] Bachmayr-Heyda A, Reiner AT, Auer K, Sukhbaatar N, Aust S, Bachleitner-Hofmann T, Mesteri I, Grunt TW, Zeillinger R, Pils D (2015). Correlation of circular RNA abundance with proliferation–exemplified with colorectal and ovarian cancer, idiopathic lung fibrosis, and normal human tissues. Scientific reports.

[7] Qin M, Liu G, Huo X, Tao X, Sun X, Ge Z, Yang J, Fan J, Liu L, Qin W (2016). Hsa_circ_0001649: A circular RNA and potential novel biomarker for hepatocellular carcinoma. Cancer Biomark A.

[8] Nair AA, Niu N, Tang X, Thompson KJ, Wang L, Kocher JP, Subramanian S, Kalari KR (2016). Circular RNAs and their associations with breast cancer subtypes. Oncotarget.

[9] Li P, Chen S, Chen H, Mo X, Li T, Shao Y, Xiao B, Guo J (2015). Using circular RNA as a novel type of biomarker in the screening of gastric cancer. Clin Chim Acta.

[10] Zhao ZJ, Shen J (2017). Circular RNA participates in the carcinogenesis and the malignant behavior of cancer. RNA Biol.

[11] Jeanson A, Boyer A, Greillier L, Tomasini P, Barlesi F (2019). Therapeutic potential of trametinib to inhibit the mutagenesis by inactivating the protein kinase pathway in non-small cell lung cancer. Expert Rev Anticancer Ther.

[12] Barlesi F, Mazieres J, Merlio JP, Debieuvre D, Mosser J, Lena H, Ouafik L, Besse B, Rouquette I, Westeel V (2016). Routine molecular profiling of patients with advanced non-small-cell lung cancer: results of a 1-year nationwide programme of the French Cooperative Thoracic Intergroup (IFCT). Lancet.

[13] Planchard D, Smit EF, Groen HJM, Mazieres J, Besse B, Helland Å, Giannone V, D’Amelio AM, Zhang P, Mookerjee B (2017). Dabrafenib plus trametinib in patients with previously untreated BRAF(V600E)-mutant metastatic non-small-cell lung cancer: an open-label, phase 2 trial. Lancet Oncol.

[14] Blumenschein GR, Smit EF, Planchard D, Kim DW, Cadranel J, De Pas T, Dunphy F, Udud K, Ahn MJ, Hanna NH (2015). A randomized phase II study of the MEK1/MEK2 inhibitor trametinib (GSK1120212) compared with docetaxel in KRAS-mutant advanced non-small-cell lung cancer (NSCLC). Ann Oncol.

[15] Gandara DR, Leighl N, Delord JP, Barlesi F, Bennouna J, Zalcman G, Infante JR, Reckamp KL, Kelly K, Shepherd FA (2017). A phase 1/1b study evaluating trametinib plus docetaxel or pemetrexed in patients with advanced non-small cell lung cancer. J Thor Oncol.

[16] Sun Y, Liu WZ, Liu T, Feng X, Yang N, Zhou HF (2015). Signaling pathway of MAPK/ERK in cell proliferation, differentiation, migration, senescence and apoptosis. J Recept Signal Transduct Res.

[17] Zhang W, Sun J, Luo J (2016). High Expression of Rab-like 3 (Rabl3) is Associated with Poor Survival of Patients with Non-Small Cell Lung Cancer via Repression of MAPK8/9/10-Mediated Autophagy. Med Sci Monit.

[18] Wu WK, Cho CH, Lee CW, Wu YC, Yu L, Li ZJ, Wong CC, Li HT, Zhang L, Ren SX (2010). Macroautophagy and ERK phosphorylation counteract the antiproliferative effect of proteasome inhibitor in gastric cancer cells. Autophagy.

[19] Liu Y, Tao X, Jia L, Cheng KW, Lu Y, Yu Y, Feng Y (2012). Knockdown of RAB25 promotes autophagy and inhibits cell growth in ovarian cancer cells. Mol Med Rep.

[20] Zhang N, Song Y, Xu Y, Liu J, Shen Y, Zhou L, Yu J, Yang M (2020). MED13L integrates Mediator-regulated epigenetic control into lung cancer radiosensitivity. Theranostics.

[21] Sun S, Wang X, Xu X, Di H, Du J, Xu B, Wang Q, Wang J (2017). MiR-433-3p suppresses cell growth and enhances chemosensitivity by targeting CREB in human glioma. Oncotarget.

[22] Shi Q, Wang Y, Mu Y, Wang X, Fan Q (2018). MiR-433-3p Inhibits Proliferation and Invasion of Esophageal Squamous Cell Carcinoma by Targeting GRB2. Cell Physiol Biochemistry.

[23] Hou XK, Mao JS (2020). Long noncoding RNA SNHG14 promotes osteosarcoma progression via miR-433-3p/FBXO22 axis. Biochem Biophys Res Commun.

[24] Liu SQ, Zhou ZY, Dong X, Guo L, Zhang KJ (2020). LncRNA GNAS-AS1 facilitates ER + breast cancer cells progression by promoting M2 macrophage polarization via regulating miR-433-3p/GATA3 axis. Bioscience Rep.

[25] Wang F, Fan M, Cai Y, Zhou X, Tai S, Yu Y, Wu H, Zhang Y, Liu J, Huang S (2020). Circular RNA circRIMS1 acts as a sponge of miR-433-3p to promote bladder cancer progression by regulating CCAR1 expression. Mol Ther Nucl Acids.

[26] Cai X, Zhang X, Mo L, Zhu J, Yu H (2020). LncRNA PCGEM1 promotes renal carcinoma progression by targeting miR-433-3p to regulate FGF2 expression. Cancer Biomark A.

